# Effective coding is key to the development and use of the WHO Essential Diagnostics List

**DOI:** 10.1016/S2589-7500(19)30196-7

**Published:** 2019-12-01

**Authors:** Jacob McKnight, Michael L Wilson, Pamela Banning, Chris Paton, Felix Bahati, Mike English, Ken Fleming

**Affiliations:** Oxford Health System Collaboration, University of Oxford, Oxford OX1 3SY, UK; African Strategies for Advancing Pathology, Denver, CO, USA; 3M Health Information Systems, Salt Lake City, UT, USA; LOINC, Regenstrief Institute, Indianapolis, IN, USA; Oxford Health System Collaboration, University of Oxford, Oxford OX1 3SY, UK; KEMRI-Wellcome Trust Research Programme, Nairobi, Kenya; Oxford Health System Collaboration, University of Oxford, Oxford OX1 3SY, UK; KEMRI-Wellcome Trust Research Programme, Nairobi, Kenya; Oxford Health System Collaboration, University of Oxford, Oxford OX1 3SY, UK

The WHO new Essential Diagnostics List (EDL) aims to provide a structure for identifying, promoting, and increasing the supply and availability of the most effective and important in-vitro diagnostics (a full list of the included diagnostics is available in the appendix). The first edition of the EDL was published in November, 2018, but many revisions are expected given both its novelty and its importance in setting goals for health policy makers globally. It has no international antecedents, but the intention is to build on the success of WHO’s well established Essential Medicines List. The EDL is not prescriptive, but is instead “expected to provide guidance and serve as a reference”.^1^ The development of the EDL is still very much in a formative phase, with much of the effort directed towards test inclusion and reasoning. If the EDL is to be useful to health policy planners, however, use of appropriate coding systems should also be an early consideration.

International information technology (IT) standards and medical codes are essential to modern medicine: by agreeing on clinical terms, the health-care ecosystem can prevent errors, improve documentation, and facilitate efficient research and quality improvement. In all health systems, patients frequently move between different health-care providers and facilities, and their health records should be available and understandable to all. In high-income countries, coding systems such as SNOMED CT, ICD11, and Logical Observation Identifiers Names and Codes (LOINC) together provide precise clinical vocabularies that allow medics to very specifically describe medical conditions and treatments in electronic health record systems. The different coding systems and data standards are the result of a long history of gradual development, with different stakeholders requiring different types of data. Data standards designed for medical billing or for aggregating data at a national or regional level might not offer the level of detail needed for data used by clinicians in their day-to-day work, viewing laboratory test results, or entering information into electronic health record systems. The diversity of these applications can result in multiple coding systems being implemented in different clinical IT systems even within the same health-care institution, which can add to complexity and increase the difficulty of linked data analysis.

The EDL is expected to have a substantial effect on the availability of the most important diagnostics in low-income and middle-income countries (LMICs) because it provides an evidence-based template to guide government purchasing, while directing scarce resources towards the most important tests. Health systems in LMICs might be assumed not to have taken up digitisation of health records yet and, therefore, coding of tests might not be considered an urgent concern. These assumptions are unfounded; despite the wide proliferation of health IT systems across Kenya, these systems have most often been developed locally and are not standardised nor interoperable (unpublished data). Therefore, to maximise the benefits of the EDL, we suggest the coding issue needs to be addressed now. Commonly used test names are often ambiguous and confusing and so without a universal coding system, organisations hoping to share data will inevitably make mistakes that could lead to medical error or reduced collaboration. Implementing coding at this phase will also enable cross referencing from multiple diagnostic suppliers with different internal codes and names.

The well recognised Principles for Digital Development insist “open standards, open data, open source, and open innovation” should be used. Although diagnostics are included in many coding systems, by far the most dominant open-source system is LOINC. LOINC is developed and supported by the non-profit Regenstrief Institute. The codes it produces are continually updated, with several volunteer groups leading different sections of coding. As such, LOINC is designed for universal use. 79 000 users in 175 countries use LOINC codes in 12 languages. Furthermore, diagnostics suppliers keep the database updated by registering new tests in the system through a so-called connectivity consortium.

Ensuring that the EDL is properly coded in LOINC provides multiple benefits. The first is patient choice: in most LMICs, medical laboratories in the public sector struggle with shortages and human resource issues that lead to patients seeking medical tests in the private sector.^2^ However, most often they are provided only with a scribbled request form, in which the technical phrasing and poor handwriting combine to ensure that patients find it difficult to compare prices and turnaround times from public and private alternative labs. By providing a specific code, a patient would know that they are comparing like for like, and comparing and contrasting providers would be easier. In turn, more rational purchasing would help to regulate the market.

The second benefit is specificity. Approximately 70% of medical laboratory errors occur before the analytical phase,^3^ which means that incorrect labelling, choosing the wrong test, or asking for the wrong sample are major sources of error. We propose that effective electronic coding helps minimise the risk of these errors by allowing (or perhaps encouraging) medics to be specific about the tests they need.

Interoperability is the third benefit of appropriate EDL coding. Networking medical laboratories together in a way that would allow smaller laboratories to send samples to more capable laboratories is a longstanding aim of global health policy makers.^4^ However, this goal is limited by ineffective communication between laboratories and between laboratory information systems. LOINC coding would allow laboratories to improve data-exchange coordination because tests would not need to be manually checked against each other as part of the process of establishing application programming interfaces. Encouraging the development of application programming interfaces between facilities is important because it could help facilitate tiering of laboratories, according to which lower-level (ie, less techinically capable) laboratories could send samples to higher-level (ie, more capable) facilities that might have additional capacity.

Finally, EDL coding can improve ordering and quality control. The EDL process will never be complete. The international list and national lists will be continually updated with new guidance for health system planners who order and supply diagnostic kits, equipment, and reagents. Appropriate coding will facilitate ordering by ensuring identification errors are reduced and allow planners to avoid potential issues with quality control.

Other coding systems feature laboratory tests, of course, and use of these systems would help achieve many of the advantages set out here and it would be sensible for the EDL team to review these systems. Nevertheless, LOINC is an open-source, free-to-use data set, in contrast to other coding systems that are proprietary. LOINC is continually updated by volunteers and is endorsed by regulatory authorities in many countries.

Regardless of the system used, increasing the availability of high-quality laboratory testing is crucial to overall improvements in clinical practice and the quality of LMIC health systems and will be an essential component in the efforts towards universal health coverage.^5^ We believe that the EDL process should involve LOINC in all aspects of the development of both international and national lists so that the community of practitioners that maintains LOINC can ensure that the coding keeps pace with the development of the EDL. In an effort to encourage this participation, we have provided with this commentary what we believe to be a first attempt to code the second edition of the EDL in LOINC, which should also facilitate the EDL’s conversion into other coding systems when needed (appendix).
